# Looking on the bright side: The relationships between flourishing and pain-related outcomes among adolescents living with chronic pain

**DOI:** 10.1177/13591053231214099

**Published:** 2023-12-15

**Authors:** Ryan D Parsons, Joanna L McParland, Sarah L Halligan, Liesbet Goubert, Melanie Noel, Abbie Jordan

**Affiliations:** 1 University of Bath, UK; 2 Glasgow Caledonian University, UK; 3 University of Cape Town, South Africa; 4 Ghent University, Belgium; 5 University of Calgary, Canada

**Keywords:** adolescence, health psychology, pain, posttraumatic growth, well-being

## Abstract

A deficits-based approach to adolescent chronic pain currently dominates the literature, to the exclusion of positive approaches, such as flourishing. Addressing this knowledge gap, this study examined the relationships between flourishing and pain-related outcomes in adolescent chronic pain. Seventy-nine adolescents aged 11–24 years were asked to complete self-report measures of three domains of flourishing and four pain-related outcomes. Correlation coefficients and four hierarchical linear regression analyses were conducted, controlling for age and gender. Flourishing mental health was associated with, and significantly contributed to explaining, anxiety and depressive symptoms, and social and family functioning impairment. Benefit finding and posttraumatic growth were each associated with social and family functioning impairment, while posttraumatic growth was also associated with anxiety and depressive symptoms. Additionally, benefit finding significantly contributed to explaining pain intensity. Study findings underscore the importance of assessing the relationships between flourishing and pain-related outcomes in adolescents with chronic pain.

## Introduction

Adolescence is a critical developmental period, marked by psychological, social, and physical changes (Eccleston et al., 2008; Jones et al., 2021). The prevalence of chronic pain (i.e. pain lasting longer than 3 months; Treede et al., 2015) increases in adolescence, with 11%–38% of this group reporting chronic pain, although prevalence rates may vary by condition (e.g. musculoskeletal pain: 4%–40%; back pain: 14%–24%; headache: 8%–83%; abdominal pain: 4%–53%) (King et al., 2011). Research has predominantly focused on negative outcomes for adolescents living with chronic pain, with reported adverse effects on physical, school, emotional, and developmental functioning, notably including high levels of anxiety, depressive symptoms, and reduced family and social functioning (Fisher and Palermo, 2016; Goubert and Trompetter, 2017; Groenewald et al., 2020; Higgins et al., 2021; Jones et al., 2021). However, evidence suggests that adolescents can also experience positive outcomes or flourish while living with chronic pain (Parsons et al., 2022).

While there has been limited previous research focused on flourishing in *adults* living with chronic pain (Gilmour, 2015; Trompetter et al., 2019), there is a dearth of research focused on adolescents in this area. This is important to consider as there may be differences in the perception, prevalence, and experience of chronic pain among these groups. For example, older individuals tend to have higher prevalence of chronic pain (Mills et al., 2019), while adolescents have been shown to display different clinically significant pain intensity rating cut points compared to adults (Hirschfeld et al., 2014). Additionally, Feinstein et al. (2017) found differences in the relationships between pain catastrophizing, pain interference, and pain intensity in younger versus older individuals.

There is no single definition or conceptualization of adolescent flourishing (Witten et al., 2019). However, within the field of mental health, flourishing is defined as the concurrent experience of high levels of psychological, emotional, and social well-being (Keyes, 2002, 2005). Flourishing has also been defined more broadly as a sense of “life going well” (Huppert and So, 2013). Drawing on this literature and aligned with our recent scoping review (Parsons et al., 2022), we build on these definitions to conceptualize flourishing in the context of chronic pain as an umbrella term to encompass positive outcomes or change despite, or as a result of, chronic pain, but extending beyond a simple reduction of negative outcomes. Therefore, flourishing may be seen as either a predictor of positive outcomes or as a positive outcome in itself (e.g. benefit finding may predict growth, while growth as a positive outcome may be considered flourishing in itself) (Parsons et al., 2022).

Elements of flourishing have been identified in previous research involving adolescents with chronic pain. In Cartwright et al. (2015), adolescents living with juvenile idiopathic arthritis demonstrated aspects of benefit finding, resilience, and personal and social growth. Additionally, Soltani et al. (2018) found that benefit finding was positively associated with anxiety, depressive symptoms, pain intensity, and pain interference in adolescents with chronic pain, although this study also found that benefit finding was negatively associated with quality of life.

Despite this evidence, there is currently no detailed understanding of the relationships that these positive aspects may have with other pain-related outcomes. The present study sought to address this knowledge gap, and aimed to extend the measurement of flourishing (using measures of flourishing mental health, benefit finding, and posttraumatic growth) to the adolescent chronic pain context, to investigate the relationships between flourishing and four pain-related outcomes. These findings may serve to supplement our understanding of flourishing in the context of adolescent chronic pain. We hypothesized that: (1) flourishing would be associated with, and significantly contribute to explaining, pain intensity, (2) flourishing would be associated with, and significantly contribute to explaining, anxiety and depressive symptoms, and (3) flourishing would be associated with, and significantly contribute to explaining, social and family functioning impairment.

## Methodology

### Participants

Recruitment and data collection occurred between May 2021 and July 2022. An a priori power analysis (two-tailed, α = 0.05, power = 0.80, effect size = 0.15) indicated a required sample size of *n* = 77 participants. Participants were based in the United Kingdom, the United States, and New Zealand, and were recruited via four approaches. These comprised (1) three United Kingdom tertiary care pediatric-specific pain clinics in the South-West of England and Scotland; (2) pain charities and social media platforms (e.g. Twitter, Facebook, university recruitment panels); (3) the National Institute for Health and Care Research Clinical Research Network Portfolio; and (4) via a database of previous study participants who indicated that they would be interested in taking part in further research studies. Study participants were required to: (1) be between 11 and 24 years old (congruent with a recent extended definition of adolescence; Sawyer et al., 2018); (2) be able to provide informed consent/assent; (3) be living with recurring or persistent chronic pain (minimum duration of 3 months); (4) possess the cognitive and English language ability to complete the measures; and (5) have access to a device with internet connectivity.

### Measures

#### Demographic information

Participants were asked to self-report on standard demographic information relating to their gender, age, ethnicity, education, and occupation.

#### Pain characteristics

Participants were asked to complete self-report measures relating to key characteristics of their chronic pain condition and its management, including the following pain-related questions: “How would you rate your level of pain over the past week?” (10-point numerical scale; 1 = No pain, 10 = Worst pain ever) (Varni et al., 1987; Von Baeyer et al., 2009); “Where is the pain usually located?” (Multiple choice fields); “How long have you had your pain symptoms?” (Text boxes to enter years and months); “Do you have a diagnosis for your pain condition?” (Multiple choice fields); “Are you receiving treatment for your pain symptoms?” (Multiple choice fields); “Please briefly describe this treatment” (text box); and “How long did you previously receive treatment?” (Text boxes to enter years and months).

#### Mental Health Continuum—Short Form

Flourishing in mental health was measured using the Mental Health Continuum—Short Form (MHC-SF; Keyes, 2009) that consists of 14 self-report items rated on a 6-point Likert scale (1 = never, 6 = everyday), to indicate the frequency of well-being that participants had experienced in the past month. The scale measures three key facets of well-being under two categories, namely hedonic well-being and positive functioning. Hedonic well-being consists of items 1–3 that measure emotional well-being, while positive functioning consists of items 4–8 that measure social well-being and items 9–14 that measure psychological well-being. To be categorically diagnosed with flourishing mental health, individuals must provide a rating of “every day” or “almost every day” for at least one of the three signs of hedonic well-being and six of the 11 signs of positive functioning, during the past month. The measure can also be summed using continuous scoring (0–70 range) with a higher score indicative of increased well-being (Keyes, 2009). An example item measuring emotional well-being is: “In the past month, how often did you feel interested in life?,” while an example item measuring psychological well-being is: “In the past month, how often did you feel that you liked most parts of your personality?.” An example item measuring social well-being is: “In the past month, how often did you feel that you had warm and trusting relationships with others?.” The MHC-SF scale had excellent internal consistency in our sample (overall: α = 0.92; younger adolescents (11–18 years): α = 0.93; older adolescents (19–24 years): α = 0.91), and has shown good internal consistency and construct validity in previous adolescent (Keyes, 2006: sample age 12–18 years) and adult (Gilmour, 2015; Keyes, 2006; Lamers et al., 2011; Trompetter et al., 2019) samples.

#### Benefit and Burden Scale for Children

We used the Benefit and Burden Scale for Children (BBSC; Currier et al., 2009) to measure potential benefit finding in our sample. This 20-item self-report scale is a revision of the Benefit Finding Scale (Phipps et al., 2007), with negatively worded “burden” items added in order to avoid predominantly socially desirable positive responses by participants (Currier et al., 2009). Items are rated on a 5-point Likert scale (1 = not at all, 5 = very much), with higher ratings indicative of increased benefit or burden. Guided by methodology in Soltani et al. (2018) and due to the positive focus of our study, we opted to solely make use of the benefit finding items of the scale in our data analysis. We adjusted the introduction and instructions preceding the measure to apply to adolescents with chronic pain as follows: “We know that having chronic pain can be very hard on young people, but you may also find some positive things about having chronic pain. Below is a list of some things, good and bad, that might happen to young people or change in their life, because of having chronic pain. Please read each statement carefully and select an option to show how much these things have happened to you since you have had chronic pain.” Example benefit finding items are: “Having had my chronic pain has helped me to become a stronger person,” “Having had my chronic pain has helped me learn who my real friends are,” and “Having had my chronic pain has taught me what is really important in life.” Internal consistency of the benefit finding subscale was very good for our sample (overall: α = 0.87; 11–18 years: α = 0.89; 19–24 years: α = 0.83) and evidence for the construct validity of the scale has been found in another adolescent study (Currier et al., 2009: sample age 8–18 years).

#### Posttraumatic Growth Inventory for Children—Revised

The Posttraumatic Growth Inventory for Children—Revised (PTGI-C-R; Kilmer et al., 2009) is a 10-item self-report scale used to measure positive changes in children and adolescents following trauma. This measure is a shortened adaptation of the Posttraumatic Growth Inventory for Children (PTGI-C; Cryder et al., 2006) that uses items suited to child and adolescent respondents. This measure has previously been used to assess posttraumatic growth in adolescents following natural disasters (Hafstad et al., 2011; Yu et al., 2010) and cancer diagnosis (Barakat et al., 2006; Yaskowich, 2003). We adjusted the introduction and instructions preceding the measure to ensure relevance for adolescents with chronic pain as follows: “For each of these, please tell us how much has changed in your life from having chronic pain.” Respondents were required to indicate their perceived level of change on a 4-point Likert scale (0 = No change, 4 = A lot), with a higher score indicating higher perceived growth. Example items are: “I learned how nice and helpful some people can be,” “I can now handle big problems better than I used to,” and “I know what is important to me better than I used to.” This measure had very good internal consistency for our sample (overall: α = 0.81; 11–18 years: α = 0.80; 19–24 years: α = 0.82) and has been shown to be a valid scale to assess posttraumatic growth in adolescents (Vloet et al., 2017).

#### Revised Child Anxiety and Depression Scale—Short Version

The Revised Child Anxiety and Depression scale (RCADS25; Ebesutani et al., 2012) is a 25-item shortened version of the Revised Child Anxiety and Depression Scale (RCADS; Chorpita et al., 2000), used to assess anxiety and depression symptoms in children and adolescents. Items are rated on a 4-point Likert scale (0 = Never, 3 = Always) to measure frequency of occurrence of symptoms. Example items are: “I feel sad or empty” and “I feel afraid that I will make a fool of myself in front of people.” Total scores of the measure are suggested to be converted into T-scores using specific equations provided, which account for age (represented by school grade) and gender differences, and indicate ranges of low, medium, and high severity of symptoms. However, we opted to sum scores of relevant anxiety and depression items instead, as higher scores on the measure still provide useful data and indicate greater degrees of symptom severity. We opted to adopt this approach as we did not have a school grade to enter into the equation for our older participants and furthermore, the options for gender differences provided only allowed for binary distinctions (male and female) and did not take other gender identifiers into account. Additionally, our research aims were to investigate relationships between quantity of anxiety and depression and flourishing predictors, so clinical distinctions between varying levels of these constructs were unnecessary. This shortened version of the RCADS measure has shown sufficient validity to assess anxiety and depression in adolescents (Klaufus et al., 2020: sample age 8–18 years), and internal consistency of this measure was excellent in our sample (overall: α = 0.92; 11–18 years: α = 0.91; 19–24 years: α = 0.94).

#### Bath Adolescent Pain Questionnaire

The Bath Adolescent Pain Questionnaire (BAPQ; Eccleston et al., 2005) is a self-report measure used to assess the impact of adolescent chronic pain on seven domains of functioning. Items are rated on a 5-point frequency scale (0 = Never, 4 = Always) to indicate perceived levels of positive and negative functioning. Positive items are reverse scored so that a higher total score indicates more impaired functioning. The BAPQ has found to be reliable and valid to assess adolescents with chronic pain (Eccleston et al., 2005: sample age 11–18 years). We used two sub-scales of the BAPQ; the social functioning and family functioning subscales. The Social Functioning subscale is a 9-item self-report measure that provides an assessment of social functioning impairment in adolescents with chronic pain. Example items are: “I go out and meet friends” and “I feel distant from my friends.” A score of 0 for this subscale indicates no impairment, while a score of 36 indicates maximum impairment due to chronic pain. Internal consistency of this subscale was very good (overall: α = 0.84; 11–18 years: α = 0.87; 19–24 years: α = 0.81). The Family Functioning subscale is a 12-item scale to assess the impact of adolescent chronic pain on family functioning impairment. Example items are: “Family life is stressful,” “My family is happy,” and “Family activities get interrupted by my pain.” A score of 0 for this subscale indicates no impairment, while a score of 48 indicates maximum impairment due to chronic pain. Internal consistency of this subscale was very good in our sample (overall: α = 0.84; 11–18 years: α = 0.85; 19–24 years: α = 0.83).

### Procedure

All participant information sheets, consent statements, demographic items, and quantitative measures were administered using Qualtrics online survey software (Qualtrics, Provo, UT, 2020; https://www.qualtrics.com). Potential participants were provided with a weblink to a Qualtrics hosted webpage where they were able to access the participant information sheet and provide informed consent (or informed parental consent and assent if under 16 years of age) before beginning the study. Following this, participants were asked to provide standard demographic information and answer questions relating to their pain characteristics, before gaining access to the measures of flourishing and pain outcomes. Detailed instructions preceded each measure to ensure that participants understood how to complete them correctly. Following completion of all measures, participants were subsequently debriefed via Qualtrics and emailed an online e-voucher to the value of £5 (United Kingdom) to thank them for their time and participation. The full protocol for this study was registered on the Open Science Framework database: https://osf.io/xtnkz/?view_only=8586a393c9ab450eaa42cc1ac7a0269d

### Data analysis

The Statistical Program for Social Sciences (SPSS v. 26; IBM Statistics) was used for all statistical analyses. Prior to analyses, data were examined to assess normality and search for potential outliers using univariate and graphical analyses. Descriptive statistics were subsequently calculated to analyze the sample characteristics and calculate mean scores for key variables. Bivariate Pearson correlation coefficients were then calculated to compare relationships between key variables in order to assess for any significant correlations, with the exception of correlations involving the *pain intensity* measure, which was calculated using Spearman’s rank correlation coefficient due to this being an ordinal variable. Following this, four hierarchical linear regression analyses were conducted to investigate the association between independent variables (i.e. flourishing mental health, benefit finding, and posttraumatic growth) and the following outcomes: (1) pain intensity; (2) anxiety and depressive symptoms; (3) social functioning impairment; and (4) family functioning impairment. The covariates of age and gender (dummy encoded before analysis) were entered into the first step of all regression models, with flourishing independent variables simultaneously entered into step 2 of each model. All correlational and regression analyses were conducted using two-tailed hypothesis testing with an alpha level of 0.05.

## Results

### Descriptive statistics

In total, 79 adolescents (66 female, 8 male, 1 trans male, 2 non-binary, 2 not disclosed) completed the study measures. See Appendix A for full participant characteristics. Participants were predominantly White (93.7%) and were between the ages of 11 and 24 years (*M* = 18.05, SD = 2.98). Participants had lived with chronic pain from 5.04 months to 20 years (*M* = 5.48, SD = 3.99). Self-reported pain intensity for the past week ranged from 3 to 10 on a Likert scale (*M* = 6.19, SD = 1.84). Common chronic pain conditions of study participants included musculoskeletal pain, Complex Regional Pain Syndrome, fibromyalgia, migraine, and juvenile arthritis. In total, 56 participants (70.9%) were currently receiving some form of treatment, while 13 participants (16.5%) had previously received treatment and 10 participants (12.6%) had not received any treatment for their pain condition. Based on Keyes (2009) categorical scoring guidance, a total of 16 of our participants (20.25%) were categorized as having flourishing mental health, indicating that they had provided a rating of “every day” or “almost every day” for at least one of the three signs of hedonic well-being and six of the 11 signs of positive functioning, during the past month. This categorization has previously been used in studies investigating adult chronic pain (Gilmour, 2015; Trompetter et al., 2019). Descriptive statistics for all measures are shown in Table 1.

**Table 1.** Descriptive statistics of self-report measures.

	Minimum	Maximum	Mean	SD
MHC-SF (Flourishing Mental Health)	9	60	36.61	13.70
BBSC (Benefit Subscale)	12	47	31.49	8.93
PTGI (Post Traumatic Growth)	0	28	12.00	5.99
RCADS (Anxiety & Depressive Symptoms)	5	68	33.34	13.92
BAPQ (Social Functioning Subscale)	7	33	18.99	6.37
BAPQ (Family Functioning Subscale)	5	45	20.42	7.86

### Correlation coefficients between key variables

Correlation coefficients were calculated to investigate relationships between flourishing variables and four pain outcome variables (see Table 2 for a correlation matrix). Flourishing in mental health was positively associated with benefit finding (*r* = 0.56, *p* < 0.001) and posttraumatic growth (*r* = 0.47, *p* < 0.001) and negatively associated with anxiety and depressive symptoms (*r* = −0.58, *p* < 0.001), social functioning impairment (*r* = −0.59, *p* < 0.001), and family functioning impairment (*r* = −0.47, *p* < 0.001). Benefit finding was positively associated with posttraumatic growth (*r* = 0.80, *p* < 0.001) and negatively associated with social functioning impairment (*r* = −0.32, *p* = 0.004) and family functioning impairment (*r* = −0.25, *p* = 0.024). Posttraumatic growth was negatively associated with anxiety and depressive symptoms (*r* = −0.25, *p* = 0.026), social functioning impairment (*r* = −0.35, *p* = 0.002), and family functioning impairment (*r* = −0.26, *p* = 0.024). Levels of pain intensity and pain duration were not significantly associated with any of our key flourishing variables in the correlations.

**Table 2.** Correlation coefficients between key variables.

	1	2	3	4	5	6	7	8	9
1. Age	—								
2. Pain duration	0.22	—							
3. Flourishing mental health	−0.14	0.08	—						
4. Benefit finding	0.07	0.14	0.56**	—					
5. Post traumatic growth	0.07	0.12	0.47**	0.80**	—				
6. Anxiety and depressive symptoms	0.01	−0.11	−0.58**	−0.22	−0.25*	—			
7. Social functioning impairment	0.07	−0.02	−0.59**	−0.32**	0.35**	0.57**	—		
8. Family functioning impairment	0.06	0.00	−0.47**	−0.25*	−0.26*	0.55**	0.40**	—	
9. Pain intensity	−0.05	0.10	0.00	0.14	0.04	0.28*	0.33**	0.12	—

** Correlation is significant at the 0.01 level (2-tailed).

* Correlation is significant at the 0.05 level (2-tailed).

### Hierarchical linear regression

Results of our hierarchical linear regression models are shown in Table 3. Relationships between independent variables and variance inflation factor (VIF; used to indicate strong linear relationship between predictors in SPSS) were not sufficiently high to indicate problems with collinearity. This was determined as all VIFs were less than 10 and the average of VIFs was not substantially greater than 1 (Field, 2018). The residuals were normally distributed in each of the regression models. When controlling for age and gender, flourishing mental health was the only independent variable that significantly contributed to explaining anxiety and depressive symptoms, with higher flourishing mental health indicating lower levels of anxiety and depressive symptoms (β = −0.71, *p* < 0.001). The total model accounted for 39% of the variance in anxiety and depressive symptoms (*R*² change = 0.35, *F* = 5.56, *p* < 0.001). Flourishing mental health was also the only independent variable that significantly contributed to explaining social functioning impairment, with higher flourishing mental health indicating lower social functioning impairment (β = −0.66, *p* < 0.001). This total model accounted for 40% of the variance in social functioning impairment (*R*² change = 0.38, *F* = 5.79, *p* < 0.001). Furthermore, flourishing mental health was also the only independent variable that significantly contributed to explaining family functioning impairment, with higher flourishing mental health indicating lower family functioning impairment (β = −0.46, *p* = 0.001). This total model accounted for 28% of the variance in family functioning impairment (*R*² change = 0.20, *F* = 3.47, *p* = 0.001). Although the *R*-squared change in step 2 of the pain intensity hierarchical regression was non-significant, the independent variable of benefit finding did significantly contribute to explaining variability in the pain intensity outcome measure, with higher benefit finding indicating higher pain intensity (β = 0.42, *p* = 0.04).

**Table 3.** Hierarchical linear regression models.

Outcome variable	Model	Predictor	*F*	Unstandardized β	Standardized β	*R* ^2^	*R*^2^ change	LLCI	ULCI	VIF
Pain intensity	1	Age	0.98	−0.08	−0.13	0.06	0.06	−0.23	0.06	1.08
		Gender (male)^a^		−1.04	−0.17			−2.47	0.38	1.09
		Gender (nonbinary)^a^		−1.94	−0.17			−4.58	0.70	1.01
		Gender (trans male)^a^		0.56	0.03			−3.15	4.26	1.01
		Gender (unspecified)^a^		−0.82	−0.07			−3.46	1.81	1.00
	2	Age	1.19	−0.10	−0.16	0.12	0.06	−0.25	0.05	1.13
		Gender (male)		−0.89	−0.15			−2.32	0.54	1.11
		Gender (nonbinary)		−2.00	−0.17			−4.62	0.62	1.01
		Gender (trans male)		0.11	0.01			−3.61	3.82	1.03
		Gender (unspecified)		−0.94	−0.08			−3.72	1.84	1.14
		MHC-SF		−0.02	−0.16			−0.06	0.02	1.72
		Benefit finding		0.09	0.42***			0.00	0.17	3.25
		Post traumatic growth		−0.08	−0.25			−0.19	0.04	2.82
Anxiety and depression	1	Age	0.54	−0.01	0.00	0.04	0.04	−1.12	1.11	1.08
		Gender (male)		−3.26	−0.07			−14.20	7.67	1.09
		Gender (nonbinary)		4.37	0.05			−15.90	24.64	1.01
		Gender (trans male)		14.87	0.12			−13.54	43.28	1.01
		Gender (unspecified)		9.88	0.11			−10.33	30.09	1.00
	2	Age	5.56*	−0.51	−0.11	0.39	0.35	−1.44	0.42	1.13
		Gender (male)		−3.67	−0.08			−12.68	5.34	1.11
		Gender (nonbinary)		1.95	0.02			−14.56	18.47	1.01
		Gender (trans male)		4.46	0.04			−18.93	27.86	1.03
		Gender (unspecified)		−7.98	−0.09			−25.52	9.55	1.14
		MHC-SF		−0.72	−0.71*			−0.97	−0.47	1.72
		Benefit finding		0.44	0.28			−0.08	0.97	3.25
		Post traumatic growth		−0.36	−0.15			−1.08	0.37	2.82
Social functioning impairment	1	Age	0.26	0.15	0.07	0.02	0.02	−0.37	0.66	1.08
		Gender (male)		−0.08	0.00			−5.13	4.97	1.09
		Gender (nonbinary)		−0.77	−0.02			−10.13	8.60	1.01
		Gender (trans male)		6.23	0.11			−6.90	19.36	1.01
		Gender (unspecified)		0.51	0.01			−8.83	9.85	1.00
	2	Age	5.79*	−0.05	−0.03	0.40	0.38	−0.48	0.37	1.13
		Gender (male)		−0.70	−0.03			−4.80	3.39	1.11
		Gender (nonbinary)		−1.79	−0.04			−9.29	5.71	1.01
		Gender (trans male)		1.61	0.03			−9.02	12.24	1.03
		Gender (unspecified)		−7.83	−0.19			−15.80	0.13	1.14
		MHC-SF		−0.31	−0.66*			−0.42	−0.19	1.72
		Benefit finding		0.11	0.15			−0.13	0.35	3.25
		Post traumatic growth		−0.19	−0.18			−0.52	0.14	2.82
Family functioning impairment	1	Age	1.38	0.09	0.03	0.09	0.09	−0.52	0.70	1.08
		Gender (male)		−3.28	−0.13			−9.29	2.73	1.09
		Gender (nonbinary)		−1.89	−0.04			−13.03	9.25	1.01
		Gender (trans male)		16.61	0.24			1.00	32.22	1.01
		Gender (unspecified)		3.97	0.08			−7.13	15.08	1.00
	2	Age	3.47*	−0.08	−0.03	0.28	0.20	−0.65	0.49	1.13
		Gender (male)		−3.96	−0.15			−9.46	1.55	1.11
		Gender (nonbinary)		−2.78	−0.06			−12.87	7.31	1.01
		Gender (trans male)		12.76	0.18			−1.53	27.06	1.03
		Gender (unspecified)		−3.51	−0.07			−14.22	7.21	1.14
		MHC-SF		−0.26	−0.45**			−0.41	−0.11	1.72
		Benefit finding		0.03	0.04			−0.29	0.35	3.25
		Post traumatic growth		−0.11	−0.08			−0.55	0.34	2.82

ULCI: upper level confidence interval; LLCI: lower level confidence interval.

a Female used as reference category.

* *p* < 0.001. **p = 0.001. ***p < 0.05.

## Discussion

This study sought to examine relationships between flourishing and four pain-related outcomes in adolescent chronic pain. Flourishing was conceptualized as high levels of well-being, and positive change or outcomes. Our study findings showed that although adolescents living with chronic pain displayed low levels of flourishing mental health overall, this domain of flourishing was significantly negatively associated with all outcome variables, apart from pain intensity. The remaining domains of flourishing, namely benefit finding and posttraumatic growth, were both significantly negatively associated with social and family functioning impairment, while posttraumatic growth was also significantly negatively associated with anxiety and depressive symptoms. Additionally, flourishing mental health significantly contributed to explaining lower levels of anxiety and depressive symptoms, and lower levels of social and family functioning impairment in our hierarchical regression models, while benefit finding significantly contributed to explaining higher levels of pain intensity.

Compared with our results, Gilmour (2015) identified unusually high levels of flourishing mental health (69%) in *adults* with chronic pain, with levels of flourishing increasing with age. These findings were based on data obtained from the 2011/2012 Canadian Community Health Survey. The authors offered the explanation that sociodemographic factors, age, and survey collection measures may all contribute to differences across studies (Gilmour, 2015). Furthermore, our study did not compare flourishing scores between individuals *with* and *without* chronic pain, as compared in Gilmour (2015). Evidence from adult populations provides mixed findings on whether individuals living with chronic pain are more or less likely to flourish than those without chronic pain. While Gilmour (2015) found lower levels of flourishing mental health among those with chronic pain, Trompetter et al. (2019) found that adults living with pain were just as likely to flourish in mental health as those without. Trompetter et al. (2019) suggest that this finding may be a result of socioeconomic advantages that led to higher levels of emotional well-being and therefore, higher levels of positive mental health in their sample of Dutch participants. These socioeconomic factors need to be taken into consideration in future research, along with age, gender, and particulars of the participant’s pain conditions, as all may help to explain individual differences in the ability to flourish in the face of chronic pain.

Our findings suggest that flourishing with chronic pain is multifaceted, and that pain-related outcomes may be related to flourishing differently. This highlights the complexity of flourishing and the dearth of knowledge that we currently have regarding potential domains of flourishing. Of particular interest in our findings was an absence of significant relationships between pain duration and flourishing. Our findings regarding pain duration align with the only previous study focused on benefit finding in chronic pain, in which Soltani et al. (2018) found no significant relationship between pain duration and benefit finding. However, Phipps et al. (2007) found that time elapsed since diagnosis made a difference to benefit finding in children with cancer, with benefit finding declining as time elapsed increased. Regarding pain intensity, only benefit finding significantly contributed to explaining pain intensity. This finding is congruent with Soltani et al. (2018) who also found a significant positive association between benefit finding and pain intensity, while Gilmour (2015) found a negative association between flourishing mental health and pain intensity. Interestingly, we found no significant relationships between benefit finding and symptoms of anxiety and depression. This contrasts with Soltani et al. (2018) who found that benefit finding was positively associated with symptoms of anxiety and depression. Oncological studies suggest that context may be important to benefit finding, causing benefits to be viewed differently by individuals due to sociodemographic and economic status (Phipps et al., 2007; Tomich and Helgeson, 2004). These varied findings are indicative of the complex relationships between flourishing domains, pain intensity, and pain duration, and warrant further investigation into the interactions between these variables to identify how these interactions may be distinct from other conditions.

Although results of our posttraumatic growth measure indicate only moderate growth in this area, literature investigating posttraumatic growth in other areas has identified significant positive outcomes. For example, a study conducted by Barakat et al. (2006) found evidence of positive growth in the majority of adolescent cancer survivors 1 year post treatment in areas such as treatment of others, plans for the future, and how they think about their lives (philosophy of life). In a more pain specific study by Dirik and Karanci (2008), evidence of posttraumatic growth was found in adults living with Rheumatoid Arthritis. It is likely that further positive outcomes such as these may also result from adolescent chronic pain, but further studies are needed to appropriately investigate posttraumatic growth in this population.

There are a number of study limitations that can be addressed in future research. First, developmental changes typically associated with adolescence may result in perceived experiences of flourishing that are not necessarily linked to chronic pain. While previous work investigating flourishing in adolescents with other conditions have identified similar positive outcomes (e.g. Barakat et al., 2006), further studies comparing positive outcomes in those with and without chronic pain would be valuable to identify whether, and how, flourishing is unique to those living with chronic pain. Second, although the co-variates of age and gender did not have any significant effects on our regression models, demographic characteristics such as these may have an important influence on flourishing in individuals with chronic pain. Our participant sample was skewed toward the upper limits of our age range (*M* = 18.05 years). As adolescence is an important time characterized by many developmental changes, there may be differences in adolescents’ capacity to flourishing based on where they are in their developmental journey. Third, differences in flourishing with chronic pain based on the country and ethnicity of our participant sample was not assessed. Gobina et al. (2019) investigated the prevalence of chronic pain among adolescents from 42 different countries and reported potential variance in the pain—related characteristics of individuals across countries, while Edwards et al. (2001) found ethnic differences in pain sensitivity, tolerance, and associated disability. However, our sample was comprised predominantly of White British Female participants. This ethnicity and gender limitation is common in pediatric pain research and results in findings that are not necessarily representative or inclusive of a diverse global population (Henrich et al., 2010). Sociodemographic limitations, including personal, contextual, and social factors may affect access to tertiary care and provide additional structural and systematic drivers of pain (e.g. discrimination, invalidation, stigma), that may affect an individual’s ability to flourish. Future studies should investigate differences in flourishing in chronic pain samples based on varied sociodemographic factors, promoting inclusivity of marginalized groups in studies, to identify potential flourishing unique to varied adolescent groups. Fourth, approximately 87% of our participants had, or were currently, receiving treatment at the time of data collection. It is possible that this may positively influence participants’ perceptions of flourishing while living with chronic pain, as positive relationships with healthcare professionals and psychological interventions have both been shown to positively influence pain outcomes (Fisher et al., 2014). Likewise, it is possible that adolescents who are less distressed by their chronic pain are more likely to take part in research such as ours. Individuals who are the most distressed may be less likely to participate in survey research, which may bias results to display a more positive and optimistic outlook (Phipps et al., 2007). Therefore, further studies comparing treatment versus non-treatment groups at multiple levels of chronic pain-related disability are needed to highlight potential differences between these groups. Fifth, certain measures adopted in our study had not previously been used with chronic pain populations or those within our participant age range, and therefore, had to be adapted to suite our needs. The following percentages of our participants were above the age ranges previously validated for these measures: BBSC: 43%; PTGI-C-R: 26.5%; RCADS: 43%; BAPQ: 43%. This approach is common in novel research and we were careful to ensure that we did not adopt measures only valid for older than our lowest participant age range. However, it is necessary to highlight this limitation to the validity of our measures, to indicate that the results should be interpreted with caution. Finally, the data collection period for this study overlapped with the COVID-19 pandemic. It is possible that the consequences of restrictions implemented to curb the spread of the virus (such as increased time spent in isolation, reduced time spent with family members and peers, restricted access to healthcare, and increased dependence on others (Zambelli et al., 2022)) may have diminished participants’ ability to flourish during this time. Alternatively, data collected toward the end of pandemic-related restrictions may have bolstered participants’ perceptions of flourishing due to newfound social engagement and freedom of movement. Therefore, further studies of this kind may be useful to investigate flourishing in chronic pain in the absence of a pandemic and its potentially confounding effects.

This study design could be broadened in several ways for future research: (1) Longitudinal studies that measure flourishing and its relationships with pain outcomes at multiple time points would be extremely valuable to identify causality between variables and how flourishing may change over time; (2) Investigations of flourishing from the perspectives of those in the adolescents’ family and social environment (such as parents, teachers, peers, and siblings) would be useful to identify perceived flourishing from multiple perspectives; (3) Self-report measures may be usefully supplemented with qualitative data collection methodology (such as daily diary entries and interviews) to further enrich the data.

In conclusion, this study provides a useful contribution to the novel investigation of flourishing in adolescent chronic pain, and the relationships between flourishing and pain-related outcomes. The study findings suggest that flourishing is possible despite chronic pain, but that flourishing is multifaceted, with various domains that are related to pain outcomes differently. This work provides a foundation for future research in this area that may be used to promote positive-focused approaches to adolescent chronic pain, which draw upon existing strengths and capabilities to support the self-management of chronic pain.

## Supplemental Material

sj-docx-1-hpq-10.1177_13591053231214099 – Supplemental material for Looking on the bright side: The relationships between flourishing and pain-related outcomes among adolescents living with chronic pain Supplemental material, sj-docx-1-hpq-10.1177_13591053231214099 for Looking on the bright side: The relationships between flourishing and pain-related outcomes among adolescents living with chronic pain by Ryan D Parsons, Joanna L McParland, Sarah L Halligan, Liesbet Goubert, Melanie Noel and Abbie Jordan in Journal of Health Psychology
