# Supplementary material for: Looking on the bright side: The relationships between flourishing and pain-related outcomes among adolescents living with chronic pain
Source: J Health Psychol. 2023 Dec 15;29(8):877–90. doi: 10.1177/13591053231214099 (PMC11264544; doi:10.1177/13591053231214099)
Supplement: sj-docx-1-hpq-10.1177_13591053231214099 – Supplemental material for Looking on the bright side: The relationships between flourishing and pain-related outcomes among adolescents living with chronic pain [file sj-docx-1-hpq-10.1177_13591053231214099.docx]

|  | Minimum | Maximum | Mean | SD |
| --- | --- | --- | --- | --- |
| **Age** | 11 | 24 | 18.05 | 2.98 |
| **Pain Symptoms (years)** | .42 | 20 | 5.48 | 3.99 |
| **Pain Intensity** | 3 | 10 | 6.19 | 1.84 |
|  |  |  |  |  |
| **Gender** | **N** | **%** |  |  |
| Female | 66 | 83.5 |  |  |
| Male | 8 | 10.1 |  |  |
| Non binary | 2 | 2.5 |  |  |
| Transgender male | 1 | 1.3 |  |  |
| Undisclosed | 2 | 2.5 |  |  |
|  |  |  |  |  |
| **Ethnicity** |  |  |  |  |
| White | 74 | 93.7 |  |  |
| Asian or Asian British | 3 | 3.8 |  |  |
| Mixed ethnicity | 2 | 2.5 |  |  |
|  |  |  |  |  |
| **Pain Location** |  |  |  |  |
| Pain in multiple sites | 43 | 50.6 |  |  |
| Pain in one or more upper limbs (e.g. arm/s) | 2 | 2.4 |  |  |
| Pain in one or more lower limbs (e.g. leg/s) | 22 | 25.9 |  |  |
| Abdominal pain (e.g. tummy ache) | 3 | 3.5 |  |  |
| Back pain | 3 | 3.5 |  |  |
| Head pain (e.g. headache/migraine) | 6 | 7.1 |  |  |
|  |  |  |  |  |
| **Treatment** |  |  |  |  |
| Currently receiving treatment | 56 | 70.9 |  |  |
| Previously received treatment | 13 | 16.5 |  |  |
| No treatment | 10 | 12.7 |  |  |

**APPENDIX A**: Demographic characteristics of study participants.
